# New Route Synthesis of Thiadiazoles, Bisthiadiazoles, Thiadiazolotriazines, and Pyrazolothiadiazoles Based on Hydrazonoyl Halides and Dihydrazinylthiadiazole

**DOI:** 10.3390/molecules22020336

**Published:** 2017-02-21

**Authors:** Abdelwahed R. Sayed, Shar Saad Al-Shihry

**Affiliations:** 1 Department of Chemistry, Faculty of Science, King Faisal University, Hofuf 31982, Saudi Arabia; sshihry@kfu.edu.sa; 2 Department of Chemistry, Faculty of Science, Beni-Suef University, Beni-Suef 62514, Egypt

**Keywords:** hydrazonoyl, dihydrazinylthiadiazole, hydrazinecarbodithioate, thiadiazoles

## Abstract

Synthesis and characterization of new thiadiazoles, bisthiadiazoles from the reaction of mono- and di-hydrazonoyl halides with various hydrazinecarbodithioate derivatives were studied. Treatment of hydrazonoyl halides with 2,5-dihydrazinyl-1,3,4-thiadiazole afforded new bistriazines containing thiadiazole; we also examined the reaction of 2,5-dihydrazinyl-1,3,4-thiadiazole with active methylene compounds to afford new pyrazoles containing thiadiazole compounds. The new synthesized compounds were identified by elemental analysis and various spectral data (Fourier transform infrared spectroscopy, mass spectrometry, ^1^H and ^13^C nuclear magnetic resonance).

## 1. Introduction

Hydrazonoyl halides are useful for the synthesis of assorted and various heterocyclic derivatives [1,2,3,4]. Treatment of hydrazonoyl halides with dithioate derivatives in dioxane and in the presence of base gave the thiadiazole derivatives [5]. Thiadiazoles are heterocyclic organic compounds with a comprehensive range of biological activities, such as anticancer [6], antivirus [7], antimicrobial [8], and anti-inflammatory [9]. Heterocycles are used in analytical chemistry [10] and have pharmaceutical properties [11]. Thiadiazoles are described and quantum chemistry is used to elucidate the chemical reactions in [12]. Studying the aromaticity of thiadiazoles via various quantitative methods is reported in [13,14].

2,5-Dimercapto-1,3,4-thiadiazole derivatives and lubricants containing them are reported in [15]. Thiadiazoles are synthesized to study biological activity in [16,17]. Thiadiazoles carrying electron-donating methyl or hydroxy on one or both of the rings have been studied in complexation with different metals [18]. Thiadiazoles are prepared for use in medicinal chemistry [19,20,21]. Diaminothiadiazoles reacted with diacidchlorides gave polyamides compounds in [22]. Treatment oxadiazolylphenylthiourea with hydrazonoyl halides gave oxadiazolylimino thiadiazoles [23].

Ferrocene is used as a multi-nuclear substance possessing the properties of both organometlallic and coordination complexes. 

Herein, we synthesized thiadiazoles, bisthiadiazoles, thiadiazolotriazines and pyrazolothiadiazoles based on hydrazonoyl halides and 2,5-dihydrazinyl-1,3,4-thiadiazole. The new final products will be identified by different techniques, such as elemental analysis, Fourier transform infrared spectroscopy (FT-IR), nuclear magnetic resonance (NMR), mass spectrometry (MS), and alternate synthesis whenever possible.

## 2. Results and Discussion

**Scheme 1:**
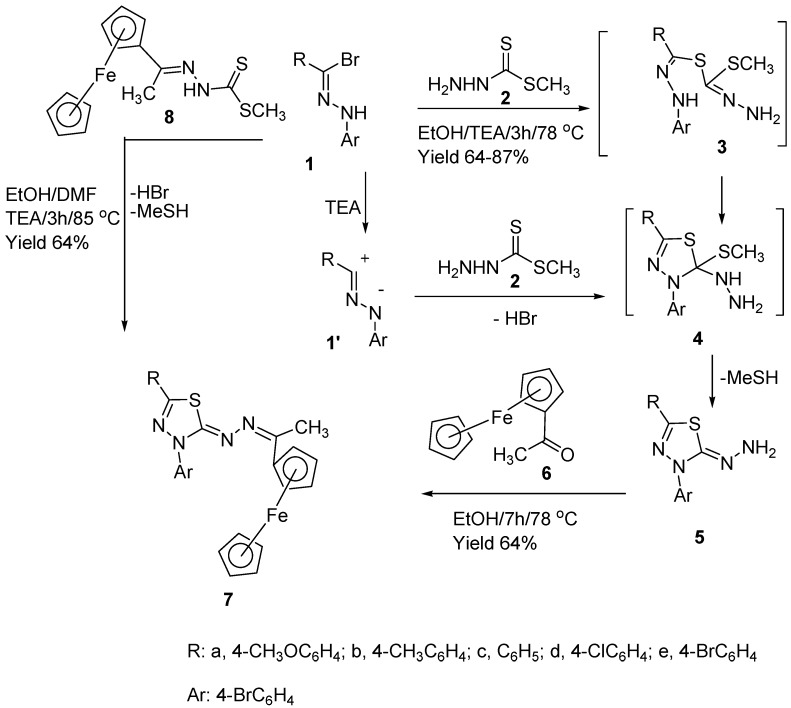
Synthesis of thiadiazole derivatives. TEA: triethylamine.

This work is a continuation of our active research in the area of hydrazonoyl halides and their reactions with different moieties, reported in [2,3]. These principles were extended in the present paper. Thus, hydrazonoyl halide **1** [24] was reacted with methyl hydrazinecarbodithioate **2** [25] in ethanol and in the presence of triethylamine under heating until complete elimination of methanethiol. The reaction mixture gave a single isolated product in each case **5a**–**e** monitored by thin layer chromatography (TLC). The formation of the final products can be explained by stepwise mechanism involving nucleophilic substitution reaction to give acyclic thiohydrazonate ester **3**, which undergoes intramolecular cyclization to yield the spirothiadiazole intermediate **4**, which was followed by elimination of methanethiol in order to give the final products **5a**–**e** or via 1,3-dipolar cycloaddition of nitrilimine (generated in situ from hydrazonoyl halides in the presence of triethylamine) to C=S of **2**, which was followed by elimination of methanethiol to give the final products **5a**–**e**. The final products were elucidated on the basis of spectral data and elemental analysis as depicted in Scheme 1. The infrared (IR) spectrum of **5a**–**e** showed absorption bands for NH_2_ group around 3333–3220 cm^−1^. In addition, the ^1^H-NMR spectrum for **5** showed signals attributed to the NH_2_ protons 5.72–5.70 ppm, as depicted in Scheme 1.

Treatment of hydrazonoyl halides **1** with methyl 2-(1-ferrocenylethylidene)hydrazine-carbodithioate **8** [26] in boiling ethanol/dimethylformamide in the presence of triethylamine under reflux afforded **7**. The assigned structure 2-(1-ferrocenylethylidenehydrazono)-3-(4-bromophenyl)-5-methoxyphenyl)-2,3-dihydro-1,3,4-thiadiazole **7** was further confirmed by alternate synthesis via the reaction of thiadiazoles **5** with the acetylferrocene **6** in ethanol, giving thiadiazole **7**, and was an identical product in all respects (mp, mixed mp, and spectra) (Scheme 1) with that obtained above from the reaction of **1a** with **8**. 

**Scheme 2:**
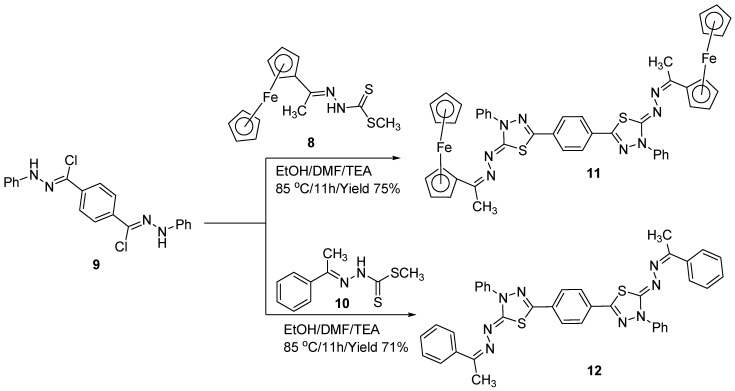
Synthesis of bisthiadiazole derivatives.

Analogously, novel compounds **11** and **12** were prepared via nucleophilic substitution reaction of 1,4-diphenylterephthalohydrazonoyl dichloride **9** [27] with methyl 2-(1-ferrocenylethylidene)hydrazinecarbodithioate **8** or methyl 2-(1-phenylethylidene)hydrazinecarbodithioate **10** [28] in EtOH/DMF and in the presence of triethylamine acting as base to give the final products **11** and **12** in good yields, as depicted in Scheme 2. The final products **11** and **12** gave a satisfactory elemental analysis and spectroscopic data (IR, NMR, and MS) consistent with their assigned structures (Scheme 2). The IR spectra of products **11** and **12** indicate the absence of NH at 3300 cm^−1^ (NH).

**Scheme 3:**
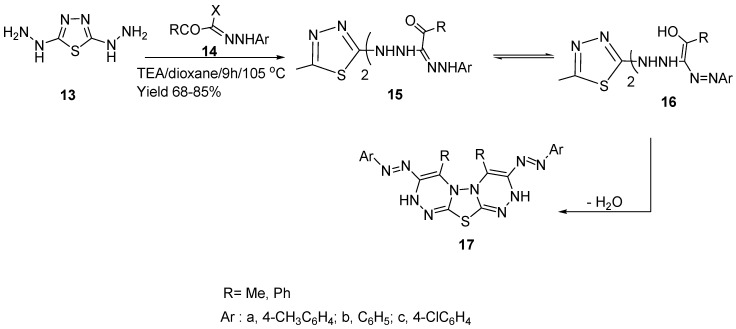
Synthesis of thiadiazolotriazines.

Pleasingly, reaction of 2,5-dihydrazinyl-1,3,4-thiadiazole **13** [29] with hydrazonoyl bromide **14** [30] in dioxane in the presence of trimethylamine as a base under reflux conditions proceeded smoothly to afford **17**; it is suggested that the reaction starts with the formation of hydrazide **15** followed by cyclization to give the product **17** via elimination of water molecule as depicted in Scheme 3. The compounds were characterized by elemental analysis and spectral data (IR, MS, ^1^H-NMR spectra) .

**Scheme 4:**
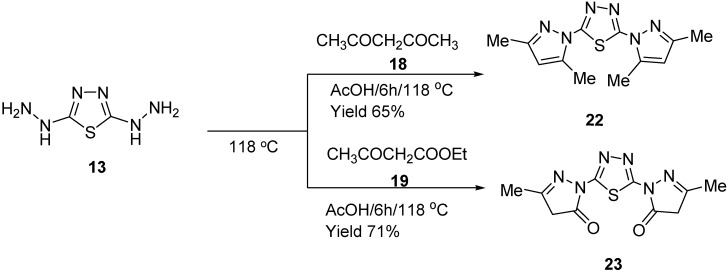
Synthesis of pyrazolothiadiazoles.

Finally, our study was extended to the reaction of 2,5-dihydrazinyl-1,3,4-thiadiazole **13** with acetylacetone **18** or ethyl acetoacetate **19** in glacial acetic acid to synthesize compounds **22** and **23**, respectively (Scheme 4). The structures of final products **22** and **23** were confirmed on the basis of spectroscopic data and elemental analyses (see Experimental Section).

## 3. Experimental Section

All melting points were determined on an electrothermal apparatus and are uncorrected. IR spectra were recorded (KBr discs) on a Shimadzu FT-IR 8201 PC spectrophotometer (Shimadzu, Tokyo, Japan). ^1^H-NMR spectra were recorded in CDCl_3_ and (CD_3_)_2_SO solutions on a Varian Gemini 300 MHz spectrometer (Agilent, Palo Alto, CA, USA), and chemical shifts are expressed in δ units using tetramethylsilane (TMS) as an internal reference. Mass spectra were recorded on a Shimadzu GC-MS QP 1000 EX instrument. Elemental analyses were carried out at the Microanalytical Canter of Cairo University. 

### 3.1. Synthesis of Thiadiazoles *(**5a**–**e**)*

To a solution of hydrazonoyl bromides **1a**–**e** (5 mmol) and methyl hydrazinecarbodithioate **2** (0.61 g, 5 mmol) in ethanol (40 mL) was added triethylamine (TEA) (5 mmol, 0.7 mL), and the mixture was refluxed for 3 h monitored by TLC. The resulting solids were collected and recrystallized from an appropriate solvent to give final products **5a**–**e**.

*3-(4-Bromophenyl)-2-hydrazono-5-(4-methoxyphenyl)-2,3-dihydro-1,3,4-thiadiazole* (**5a**). Pale green crystalline solid from EtOH/DMF, Yield (69%); m.p. 198 °C; IR (cm^−1^) (KBr): 3315, 3180 (NH_2_), 1604 (C=N); ^1^H-NMR (300 MHz, DMSO-*d*_6_): δ 3.73 (s, 3H, OCH_3_), 5.72 (s, 2H, NH_2_), 6.49–7.84 (m, 8H, ArH) ppm. Anal. Calcd. for C_15_H_13_BrN_4_OS: C, 47.76; H, 3.47; N, 14.85. Found: C, 47.72; H, 3.49; N, 14.81%.

*3-(4-Bromophenyl)-2-hydrazono-5-(p-tolyl)-2,3-dihydro-1,3,4-thiadiazole* (**5b**). Pale green crystalline solid from EtOH/DMF, Yield (64%); m.p. 177 °C; IR (cm^−1^) (KBr): 3300, 3205 (NH_2_), 1601 (C=N); ^1^H-NMR (300 MHz, DMSO-*d*_6_): δ 2.34 (s, 3H, CH_3_), 5.72 (s, 2H, NH_2_), 6.71–7.81 (m, 8H, ArH); ^13^C-NMR (300 MHz, DMSO-*d*_6_): δ 21.43, 126.47, 127.77, 129.48, 130.33, 132.53, 136.312, 140.53, 141.63, 150.75, and 154.91 ppm. Anal. Calcd. for C_15_H_13_BrN_4_S: C, 49.87; H, 3.63; N, 15.51. Found: C, 49.89; H, 3.61; N, 15.53%.

*3-(4-Bromophenyl)-2-hydrazono-5-phenyl-2,3-dihydro-1,3,4-thiadiazole* (**5c**). Pale yellow crystalline solid from EtOH/DMF, Yield (73%); m.p. 179 °C; IR (cm^−1^) (KBr): 3333, 3210 (NH_2_), 1600 (C=N); ^1^H-NMR (300 MHz, DMSO-*d*_6_): δ 5.71 (s, 2H, NH_2_), 6.78–7.91 (m, 9H, ArH) ppm. Anal. Calcd. for C_14_H_11_BrN_4_S: C, 48.43; H, 3.19; N, 16.14. Found: C, 48.45; H, 3.21; N, 16.11%.

*3-(4-Bromophenyl)-2-hydrazono-5-(4-chlorophenyl)-2,3-dihydro-1,3,4-thiadiazole* (**5d**). Pale yellow crystalline solid from EtOH/DMF, Yield (87%); m.p. 183 °C; IR (cm^−1^) (KBr): 3300, 3222 (NH_2_), 1610 (C=N); ^1^H-NMR (300 MHz, DMSO-*d*_6_): 5.70 (s, 2H, NH_2_), 6.81–7.96 (m, 8H, ArH) ppm. Anal. Calcd. for C_14_H_10_BrClN_4_S: C, 44.06; H, 2.64; N, 14.68. Found: C, 44.09; H, 2.68; N, 14.65%.

*3-(4-Bromophenyl)-2-hydrazono-5-(4-bromophenyl)-2,3-dihydro-1,3,4-thiadiazole* (**5e**). Pale green crystalline solid from EtOH/DMF, Yield (81%); m.p. 145 °C; IR (cm^−1^) (KBr): 3305, 3200 (NH_2_), 1604 (C=N); ^1^H-NMR (300 MHz, DMSO-*d*_6_): δ 5.70 (s, 2H, NH_2_), 6.81–8.01 (m, 8H, ArH) ppm. Anal. Calcd for C_14_H_10_Br_2_N_4_S: C, 39.46; H, 2.37; N, 13.15. Found: C, 39.44; H, 2.39; N, 13.19%.

### 3.2. Synthesis of 2-(1-Ferrocenylethylidenehydrazono-3-(4-bromophenyl)-5-methoxyphenyl)-2,3-dihydro-1,3,4-thiadiazole *(**7**)*

**Method A**: An equimolar amount of the appropriate hydrazonoyl bromides **1a** (5 mmol, 1.135 g) and methyl 2-(1-ferrocenylethylidene)hydrazinecarbodithioate **8** (5 mmol, 1.645 g) in 10 mL DMF, 30 mL ethanol, triethylamine (5 mmol, 0.7 mL); the reaction mixtures were refluxed for 7 h at boiling point monitored by TLC. The solvent was evaporated, and the residue was triturated with methanol. The formed solid was filtered and recrystallized from appropriate to give compounds **7**.

**Method B**: An equimolar amount of the appropriate 1,3,4-thiadiazoline derivatives **5a** (5 mmol, 1.885 g) and acetylferrocene **6** (5 mmol, 0.113 g) in absolute ethanol (40 mL) were refluxed for 7 h. The resulting solids were collected by cooling and crystallized from an appropriate solvent to give **7**.

*2-(1-Ferrocenylethylidenehydrazono)-3-(4-bromophenyl)-5-methoxyphenyl)-2,3-dihydro-1,3,4-thiadiazole* (**7**). Brown crystalline solid from EtOH/DMF; Yield (64%); m.p. 175 °C; IR (cm^−1^) (KBr): 3081, 3055 (C-H Ar), 1615 (C=N); ^1^H-NMR (300 MHz, DMSO-*d*_6_): δ 2.51 (s, 3H, CH_3_), 3.72 (s, 3H, OCH_3_), 4.18 (m, 5H, C_5_H_5_), 4.64 (m, 4H, C_5_H_4_), 6.99–8.32 (m, 8H, ArH’s) ppm. Anal. Calcd. for C_27_H_23_BrFeN_4_OS: C, 55.22; H, 3.95; N, 9.54. Found: C, 55.19; H, 3.91; N, 9.56%.

### 3.3. Synthesis of Bisthiadiazoles *(**11**)* and *(**12**)*

An equimolar amount of the appropriate 1,4-diphenylterephthalohydrazonoyl dichloride **9** (5 mmol, 1.91 g) and methyl 2-(1-ferrocenylethylidene)hydrazinecarbodithioate **8** (10 mmol, 3.29 g) or methyl 2-(1-phenylethylidene)hydrazinecarbodithioate **10** (10 mmol, 2.24 g) in ethanol/DMF (30/10 mL) was added triethylamine (10 mmol, 1.4 mL), dropwise were refluxed for 11 h at boiling point. The solvent was evaporated and the residue was triturated with methanol. The formed solid was filtered and recrystallized from appropriate solvent to give compounds **11** and **12**.

*1,4-Bis-(5-(1-ferrocenylethylidenehydrazono)-4-phenyl-4,5-dihydro-1,3,4-thiadiazol-2-yl)benzene* (**11**). Brown crystalline solid from EtOH/DMF; Yield (75%); >300 °C; IR (cm^−1^) (KBr): 3081, 3055 (C-H Ar), 1609 (C=N); ^1^H-NMR (300 MHz, DMSO-*d*_6_): δ 2.52 (s, 6H, 2CH_3_), 4.18 (m, 10H, 2C_5_H_5_), 4.64 (m, 8H, 2C_5_H_4_), 6.99–8.32 (m, 14H, ArH’s) ppm. Anal. Calcd. for C_46_H_38_Fe_2_N_8_S_2_: C, 62.88%; H, 4.36%; N, 12.75%. Found: C, 62.84%; H, 4.39%; N, 12.77%.

*1,4-Bis-(5-(1-(phenylethylidenehydrazono)-4-phenyl-4,5-dihydro-1,3,4-thiadiazol-2-yl)benzene* (**12**). Brown crystalline solid from EtOH/DMF; Yield (71%); >300 °C; IR (cm^−1^) (KBr): 3100, 3053 (C-H Ar), 1615 (C=N); ^1^H-NMR (300 MHz, DMSO-*d*_6_): δ 2.52 (s, 6H, 2CH_3_), 7.49–8.51 (m, 14H, ArH’s) ppm. Anal. Calcd. for C_32_H_30_N_8_S_2_: C, 68.86%; H, 4.56%; N, 16.91%. Found: C, 68.85%; H, 4.58%; N, 16.89%.

### 3.4. Synthesis of [1,3,4]Thiadiazolo[2,3-c:5,4-c']bis([1,2,4]triazines) (***17a**–**c***)

To 2,5-dihydrazinyl-1,3,4-thiadiazole **13** (5 mmol, 0.73 g) and the appropriate hydrazonoyl halides **14** (10 mmol) in dioxane (50 mL) was added triethylamine (1.4 mL, 10 mmol) at room temperature. The reaction mixture was heated under reflux until all the starting material was consumed (9 h, monitored by TLC). The solvent was evaporated and the residue was triturated with MeOH. The formed solid was filtered and recrystallized from DMF to give compounds **17**.

*4,7-Dimethyl-3-((E)-p-tolyldiazenyl)-8-(p-tolyldiazenyl)-2,9-dihydro-[1,3,4]thiadiazolo[2,3-c:5,4-c']bis([1,2,4]triazine)* (**17a**). Yellow crystalline solid from EtOH/DMF; Yield (85%); m.p. 275 °C; IR (cm^−1^) (KBr): 3259 (s, 2H, NH), 1610 (C=N); ^1^H-NMR (300 MHz, DMSO-*d*_6_): δ 2.58 (s, 6H, 2CH_3_), 3.53 (s, 6H, 2CH_3_), 6.99–7.91 (m, 8H, ArH’s), 11.14 (s, 2H, 2NH) ppm. Anal. Calcd. for C_22_H_22_N_10_S: C, 57.63%; H, 4.84%; N, 30.55%. Found: C, 57.66%; H, 4.89%; N, 30.51%.

*4,7-Dimethyl-3,8-bis(phenyldiazenyl)-2,9-dihydro-1,3,4-thiadiazolo[2,3-c:5,4-c]bis([1,2,4]triazine)* (**17b**). Red crystalline solid from EtOH/DMF; Yield (73%); m.p. 177 °C; IR (cm^−1^) (KBr): 3259 (s, 2H, NH), 1610 (C=N); ^1^H-NMR (300 MHz, DMSO-*d*_6_): δ 2.58 (s, 6H, 2CH_3_), 7.11–8.01 (m, 11H, ArH’s), 11.17 (s, 2H, 2NH) ppm; ^13^C-NMR (300 MHz, DMSO-*d*_6_): δ 25.54, 114.70, 115.45, 122.15, 123.32, 124.04, 129.47, 129.47, 130.01, 142.34 and 194.68 ppm. Anal. Calcd. for C_20_H_18_N_10_S: C, 55.80%; H, 4.21%; N, 32.54%. Found: C, 55.83%; H, 4.24%; N, 32.58%.

*3-((4-Chlorophenyl)diazenyl)-8-((E)-(4-chlorophenyl)diazenyl)-4,7-dimethyl-2,9-dihydro-[1,3,4]thiadiazolo[2,3-c:5,4-c']bis([1,2,4]triazine)* (**17c**). Yellow crystalline solid from EtOH/DMF; Yield (68%); m.p. 245 °C; IR (cm^−1^) (KBr): 3259 (s, 2H, NH), 1610 (C=N); ^1^H-NMR (300 MHz, DMSO-*d*_6_): δ 2.58 (s, 6H, 2CH_3_), 7.23–8.24 (m, 8H, ArH’s), 11.19 (s, 2H, 2NH) ppm. Anal. Calcd. for C_20_H_16_Cl_2_N_10_S: C, 48.10%; H, 3.23%; N, 28.05%. Found: C, 48.15%; H, 3.21%; N, 28.057%.

### 3.5. Synthesis of Pyrazolothiadiazole *(**22**)* and *(**23**)*

A mixture of 2,5-dihydrazinyl-1,3,4-thiadiazole **13** (5 mmol, 0.73 g) and acetylacetone **18** (10 mmol, 1.00 g) or ethyl acetoacetate **19** (10 mmol, 1.30 g) in glacial acetic acid (20 mL) was refluxed for 6 h. After cooling, the precipitate was collected by filtration and crystallized from the appropriate solvent to afford compounds **22** and **23**.

*2,5-Bis(3,5-dimethyl-1H-pyrazol-1-yl)-1,3,4-thiadiazole* (**22**). Pale yellow crystalline solid from EtOH/DMF; Yield (65%); m.p. 183 °C; IR (cm^-1^) (KBr): 3053 (C-H Ar), 1610 (C=N); ^1^H-NMR (300 MHz, DMSO-*d*_6_): δ 3.50 (s, 12H, 2CH_3_), 6.18 (s, 2H, 2 azomethine) ppm, ^13^C-NMR (300 MHz, DMSO-*d*_6_): δ 17.31, 114.61, 151.95, 163.05, 188.10 ppm. Anal. Calcd. for C_12_H_14_N_6_S: C, 52.54%; H, 5.14%; N, 30.63%. Found: C, 52.56%; H, 5.17%; N, 30.61%.

*1,1′-(1,3,4-Thiadiazole-2,5-diyl)bis(3-methy-1H-pyrazol-5(4H)-one)* (**23**). Pale yellow crystalline solid from EtOH/DMF; Yield (71%); m.p. 275 °C; IR (cm^-1^) (KBr): 3055 (C-H Ar), 1715 (C=O), 1615 (C=N); ^1^H-NMR (300 MHz, DMSO-*d*_6_): δ 3.17 (s, 6H, 2CH_3_), 3.84 (s, 4H, 2CH_2_) ppm. Anal. Calcd for C_10_H_10_N_6_O_2_S: C, 43.16%; H, 3.62%; N, 30.20%. Found: C, 43.19%; H, 3.64%; N, 30.24%.

## 4. Conclusions

In conclusion, the studied reactions provide a facile new route for synthesized thiadiazoles, bisthiadiazoles, pyrazolothiadiazoles, and thiadiazolotriazines via the utility of hydrazonoyl halides and 2,5-dihydrazinyl-1,3,4-thiadiazole. The final products were identified by different techniques, such as elemental analysis and FT-IR, NMR, mass spectrometry, and alternate synthesis whenever possible.
